# In-Silico Study of Immune System Associated Genes in Case of Type-2 Diabetes With Insulin Action and Resistance, and/or Obesity

**DOI:** 10.3389/fendo.2021.641888

**Published:** 2021-04-13

**Authors:** Basmah Medhat Eldakhakhny, Hadeel Al Sadoun, Hani Choudhry, Mohammad Mobashir

**Affiliations:** ^1^ Department of Clinical Biochemistry, Faculty of Medicine, King Abdulaziz University, Jeddah, Saudi Arabia; ^2^ Stem Cell Unit, Department of Medical Laboratory Technology, Faculty of Applied Medical Sciences, King Fahd Medical Research Center, King Abdulaziz University, Jeddah, Saudi Arabia; ^3^ Cancer and Mutagenesis Unit, Department of Biochemistry, Cancer Metabolism and Epigenetic Unit, Faculty of Science, King Fahd Medical Research Center, King Abdulaziz University, Jeddah, Saudi Arabia; ^4^ SciLifeLab, Department of Oncology and Pathology, Karolinska Institutet, Stockholm, Sweden

**Keywords:** type-2 diabetes and obesity, insulin action and resistance, immune system, enriched pathways, differentially expressed genes, TCR network, network-level understanding

## Abstract

Type-2 diabetes and obesity are among the leading human diseases and highly complex in terms of diagnostic and therapeutic approaches and are among the most frequent and highly complex and heterogeneous in nature. Based on epidemiological evidence, it is known that the patients suffering from obesity are considered to be at a significantly higher risk of type-2 diabetes. There are several pieces of evidence that support the hypothesis that these diseases interlinked and obesity may aggravate the risk(s) of type-2 diabetes. Multi-level unwanted alterations such as (epi-) genetic alterations, changes at the transcriptional level, and altered signaling pathways (receptor, cytoplasmic, and nuclear level) are the major sources that promote several complex diseases, and such a heterogeneous level of complexity is considered as a major barrier in the development of therapeutics. With so many known challenges, it is critical to understand the relationships and the shared causes between type-2 diabetes and obesity, and these are difficult to unravel and understand. For this purpose, we have selected publicly available datasets of gene expression for obesity and type-2 diabetes, have unraveled the genes and the pathways associated with the immune system, and have also focused on the T-cell signaling pathway and its components. We have applied a simplified computational approach to understanding differential gene expression and patterns and the enriched pathways for obesity and type-2 diabetes. Furthermore, we have also analyzed genes by using network-level understanding. In the analysis, we observe that there are fewer genes that are commonly differentially expressed while a comparatively higher number of pathways are shared between them. There are only 4 pathways that are associated with the immune system in case of obesity and 10 immune-associated pathways in case of type-2 diabetes, and, among them, only 2 pathways are commonly altered. Furthermore, we have presented SPNS1, PTPN6, CD247, FOS, and PIK3R5 as the overexpressed genes, which are the direct components of TCR signaling.

## Introduction

Type 2 diabetes (T2D) is a global epidemic that is strongly correlated with obesity (1, 2). Adipose tissue inflammation associated with obesity is known as a major cause for decreased insulin sensitivity in the case of T2D (1, 3, 4). There are a number of works where the crosstalk between the immune system and metabolism have been presented alongside evidence that supports the hypothesis that these diseases are interlinked and that obesity may aggravate the risk(s) of T2D (5–8). In such cases, it is critical to understand multi-level unwanted alterations such as (epi-)genetic alterations, changes at the transcriptional level, and altered signaling pathways (receptor, cytoplasmic, and nuclear level). These are the major sources that promote a number of complex diseases and such heterogeneous complexities are considered the major barrier in the development of therapeutic approaches. With such diverse challenges, it is crucial to understand and unravel the relationships and the common causes between type-2 diabetes and obesity (9–13).

So far, there are several studies where a number of factors have been investigated to understand the biological mechanism of obesity and T2D and the relationship between the two (1, 2, 14–16). T2D is mainly characterized by a progressive status of chronic disease, and inflammation of low-grade disease and inflammatory responses are triggered by many factors. The well-known factors contributing to T2D are age, metabolic syndrome, systematic low-grade inflammation, insulin resistance, islet cell autoantibodies, beta-cell dysfunction, and C-peptides (2, 17–23). Some of the factors that promote inflammation are altered functions of specific T lymphocyte cells, B lymphocytes, Th1, and Th17 (24–28). As mentioned above, T2D is also linked with obesity (29), and these diseases are also the case of metabolic disorder; there thus exists strong evidence for an immune-metabolic connection (10, 30, 31).

For this purpose, we have selected publicly available datasets of gene expression for obesity and T2D and have unraveled the genes and the pathways associated with the immune system; we have also focused on the T-cell signaling pathway and its components. We have applied a simplified computational approach to understanding differential gene expression and patterns and the enriched pathways for obesity and T2D. Furthermore, we have also analyzed the genes by using network-level understanding. In the analysis, we observe that there are fewer genes that are often differentially expressed while a comparatively higher number of pathways are shared between them. There are only 4 pathways that are associated with the immune system in the case of obesity and 10 immune-associated pathways in the case of T2D; among these, only two pathways are commonly altered. Here, we have studied the differentially expressed genes (DEGs), enriched pathways, and the connection between immune signaling pathways *via* their components between obesity and T2D and with the major focus being on the roles of TCR signaling components.

## Results

As mentioned above, we have compared immune signaling genes by using gene expression profiling of T2D and obesity for which the datasets have been collected from publicly available data. Here, the results have been presented from work from generalized analyses (i.e., DEGs and enriched pathways) to ensure precise analysis, i.e., TCR signaling.

### Gene Expression Profiling for Type-2 Diabetes and Obesity

**Figure 1:**
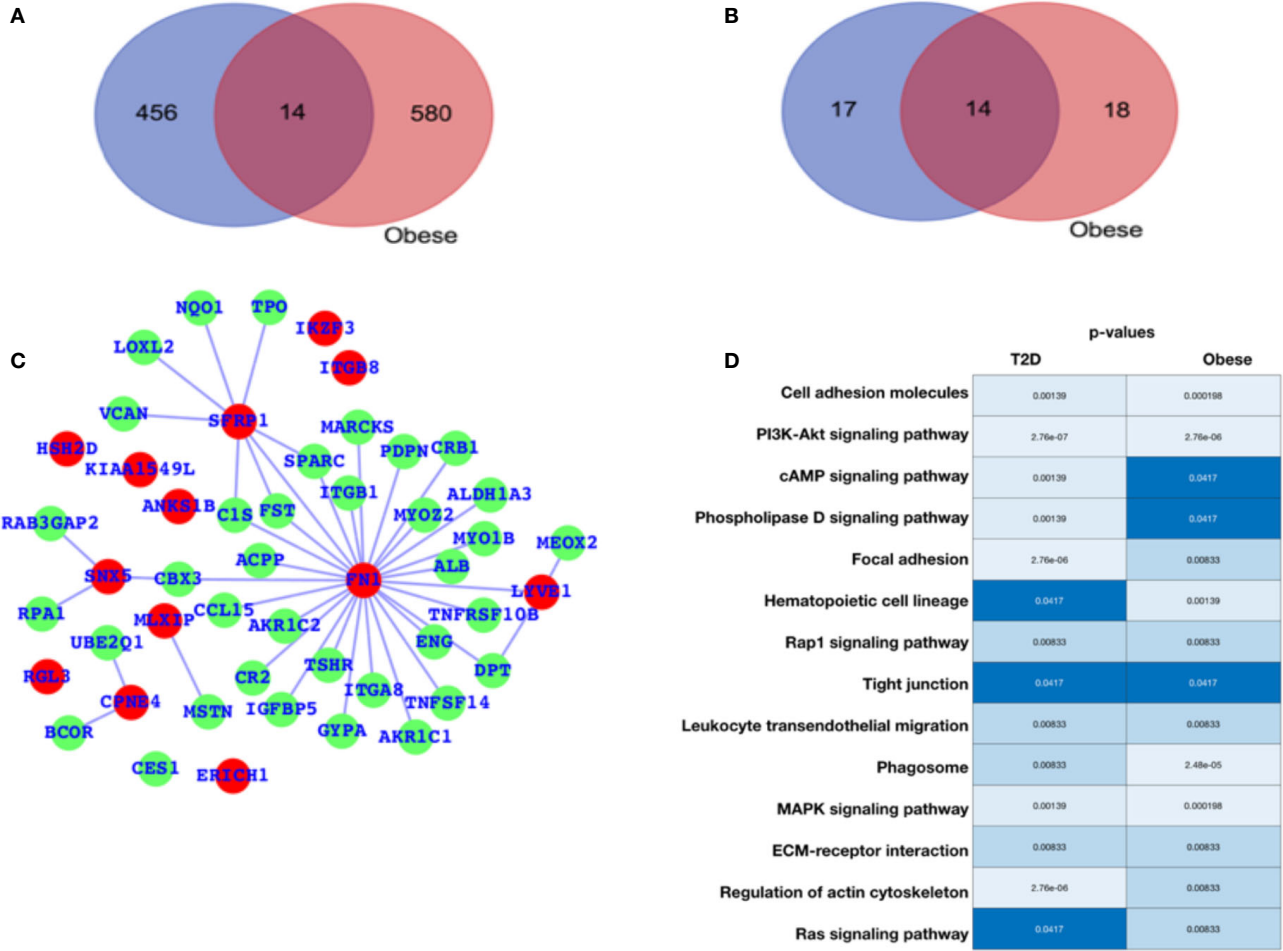
Gene expression profiling. **(A)** Venn diagram for differentially expressed genes. **(B)** Venn diagram for enriched pathways. **(C)** Network representing the DEGs common between T2D and obesity. **(D)** Commonly enriched pathways: the lighter color or lower p-value refer to higher enrichment, and the darker blue color signifies a lower p-value (close to 0.05) and thus lesser enrichment, though all these pathways are significant in terms of p-values. The p-values are mentioned in the respective color columns and rows.

For diabetes and obesity, the data have been collected from the public database, and we have performed a comparative analysis in terms of altered gene expression patterns and their respective functions. Here, we have presented the DEGs for both the cases T2D and obesity (**Figure 1A**), and we observe that there are only 12 genes that are differentially expressed in both the case; 456 are T2D-specific genes, and 580 genes are obesity-specific genes. In terms of the altered functions, they share 14 pathways, and the T2D- and obesity-specific pathways have 17 and 18 (**Figure 1B**, **Table 1**), respectively. Based on this, it appears that there are only a few genes that are differentially expressed in both cases, while in terms of biological functions, a large number of pathways are affected, which means that T2D and obesity share more biological functions in terms of alterations and their source of alterations, i.e., DEGs are not shared at a large scale. The green nodes are the DEGs that either belong to T2D or obesity cases. For this reason, we have mapped out the network for common DEGs and highlighted common DEGs in red (**Figure 1C**). Among these common DEGs, FN1, SFRP1, and SNX5 appear to be the major sources of alteration. FN1 appears to be associated with a large number of DEGs, leading to the conclusion that either FN1 is overexpressed by those genes or it promotes the overexpression of the directly associated genes. To understand the alteration in biological function, a heatmap is shown in **Figure 1D** for those pathways that are commonly enriched. For this plot, the p-values in both conditions have been used. Cell adhesion molecules, PI3K—AKT, and MAPK pathways appear highly enriched in both conditions; there is regulation of the actin cytoskeleton, cAMP, and phospholipase D; Focal adhesion is exclusively and highly enriched in the case of T2D; and hematopoietic cell lineage and phagosomes are potentially enriched in cases of obesity. There are additional pathways that are enriched in both cases but have comparatively higher *p-values*, and these pathways are the Rap1, tight junction, leukocyte transendothelial migration, ECM-receptor interaction, and Ras signaling pathways. The complete details of the fold changes of the genes are presented in **Supplementary Data S1** and **S2**.

**Table 1:** Pathways common between T2D and obesity and specific to T2D and obesity (Venn diagram outcome).

Name	Total	Pathways
T2D and Obese	14	Cell adhesion molecules (CAMs); PI3K-Akt signaling pathway; cAMP signaling pathway; Phospholipase D signaling pathway; Focal adhesion; Hematopoietic cell lineage; Rap1 signaling pathway; Tight junction; Leukocyte transendothelial migration; Phagosome; MAPK signaling pathway; ECM-receptor interaction; Regulation of actin cytoskeleton; Ras signaling pathway
T2D	17	Neurotrophin signaling pathway; Insulin signaling pathway; Vascular smooth muscle contraction; Sphingolipid signaling pathway; cGMP-PKG signaling pathway; mTOR signaling pathway; T cell receptor signaling pathway; Natural-killer-cell-mediated cytotoxicity; Jak-STAT signaling pathway; Progesterone-mediated oocyte maturation; B cell receptor signaling pathway; Oxytocin signaling pathway; Platelet activation; Thyroid hormone signaling pathway; Wnt signaling pathway; ErbB signaling pathway; Ubiquitin mediated proteolysis
Obese	18	Fatty acid metabolism; Cell cycle; Tyrosine metabolism; Thyroid hormone synthesis; Starch and sucrose metabolism; Purine metabolism; Cytokine-cytokine receptor interaction; Metabolism of xenobiotics by cytochrome P450; Retinol metabolism; Drug metabolism - cytochrome P450; Neuroactive ligand-receptor interaction; Tryptophan metabolism; p53 signaling pathway; Complement and coagulation cascades; Antigen processing and presentation; Arachidonic acid metabolism; Axon guidance; Retrograde endocannabinoid signaling

### Genes Associated With the Immune System

**Figure 2:**
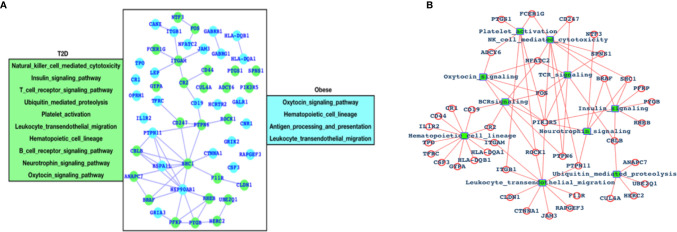
Genes associated with immune systems. **(A)** Green-colored genes belong to the immune system when the patient is suffering from T2D and cyan-colored genes belong to the immune system in case of obesity and **(B)** network of immune signaling pathways and their components (DEGs).

After analyzing the overall DEGs and all the enriched pathways, we have performed an immune-system-specific analysis for the genes and the pathways. Here, we observed that there are 10 pathways associated with the immune system in the case of T2D and four pathways in the case of obesity (**Figure 2A**). Hematopoietic cell lineage and leukocyte transendothelial migration pathways are commonly altered in both the cases T2D and obesity, and these two pathways are known to play roles in blood cell development and immune surveillance and inflammation leading to the conclusion that in both T2D and obesity, the blood cell development, immune surveillance, and the inflammation process are affected. Obesity-specific immune signaling pathways are oxytocin and antigen processing and presentation (APP). The oxytocin signaling pathway is known to exert a wide range of effects (central or peripheral), and this pathway is itself known to crosstalk with a number of critical pathways such as RhoA, GPCR, MAPK, ANP-cGMP, and NO-cGMP. APP is one of the most important parts of the entire immune system and is the process by which the protein antigen is ingested by an antigen-presenting cell (APC), partially digested into peptide fragments, and then displayed on the surface of the APC associated with an antigen-presenting molecule (MHC class I or MHC class II) for recognition by specific lymphocytes (T cells). There are eight immune-system-specific pathways in the case of T2D: NK-cell-mediated cytotoxicity, insulin signaling, TCR signaling, ubiquitin-mediated proteolysis, platelet activation BCR signaling, neurotrophin, and oxytocin signaling pathways. Almost all these pathways are major parts of the immune system. Based on this, we could conclude that in the case of T2D, there are more alterations in the gene expression pattern of those genes that are known to control major parts of the immune system, while in the case of obesity there less of an effect than T2D, but these few pathways are also known to be associated with controlling critical roles within the immune system. Furthermore, immune signaling pathways and their components have been presented in the form of a network, where hematopoietic cell lineage appears to be directly controlled by 12 highly significant DEGs; leukocyte transendothelial migration is directly controlled by 10 DEGs; ubiquitin-mediated proteolysis is controlled by 5 DEGs; the insulin signaling pathway is controlled by 6 DEGs; 3 DEGs control platelet activation; 9 DEGs control NK-cell-mediated cytotoxicity; 5 DEGs control TCR and BCR signaling; and 5 DEGs control neurotrophic signaling (**Figure 2B**).

### DEGs of T2D and TCR Signaling Network

**Figure 3:**
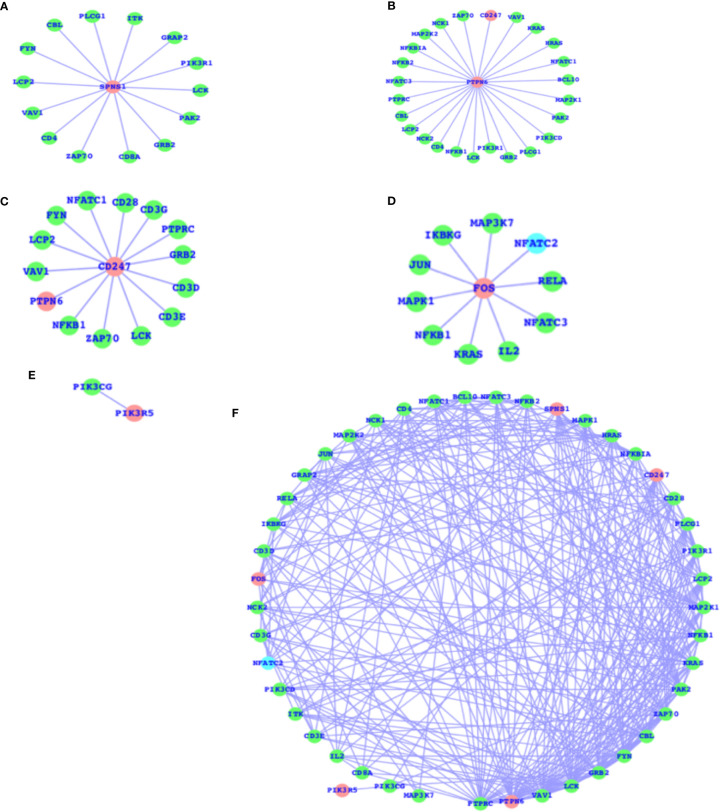
DEGs of T2D and TCR signaling network. **(A)** SPNS1 network, **(B)** PTPN6 network, **(C)** CD247 network, **(D)** FOS network, **(E)** PIK3R5 network, and **(F)** the overall association between these T2D DEGs and the TCR genes and the intra-associations.

As mentioned above, there are a number of pathways that are affected as a result of T2D and obesity; in comparison to T2D, there are only four pathways that are, due to obesity, mainly affected, and the TCR signaling pathway is not affected here. The TCR signaling pathway is affected in the case of T2D. Therefore, we have now mapped out the individual genes that are associated with the TCR signaling pathway and presented the networks for all the five genes (SPNS1, PTPN6, CD247, FOS, and PIK3R5) that are components of the TCR signaling pathway and their association with the other TCR genes (**Figures 3A–E**). These five genes have comparatively higher fold changes, i.e., the expressions and fold changes for SPNS1, PTPN6, CD247, FOS, and PIK3R5 are -5.68, -1.65, 2.72, 1.58, and 2.61, respectively, in the case of T2D. In addition, we have also presented the TCR network (**Figure 3F**) for T2D and shown the overall connectivities for all the interactors, including these five genes where PTPRC has the highest connectivity, connecting with these five genes and the majority of the interactors. PTPN6 is the second most connected, and there are more genes with much higher connectivity than SPNS1, CDC247, FOS, and PIK3R5: VAV1, LCK, GRB2, FYN, CBL, ZAP70, PAK2, KRAS, and more. The majority of these top-ranked genes in terms of connectivity are considered the core components of TCR activation and signal communication, leading to the conclusion that these core genes (SPNS1, PTPN6, CD247, FOS, and PIK3R5), which are differentially expressed, may lead to the alteration of the TCR core components and may finally potentially alter the TCR signaling process.

The SPNS1 gene is the direct component of TCR, NK-cell-mediated cytotoxicity, and FC-epsilon RI signaling pathways; PTPN6 belongs to TCR, BCR, NK-cell-mediated cytotoxicity, JAK-STAT, and the adherens junction; CD247 belongs to TCR and NK-cell-mediated cytotoxicity; and FOS belongs to TCR, BCR, TLR, MAPK, TNF, cAMP, estrogen, oxytocin, prolactin, circadian entrainment, and osteoclast differentiation. PIK3R5 alone belongs to a large number of critical pathways, such as TCR, BCR, NK cell-mediated cytotoxicity, JAK-STAT, ErbB, chemokine, phosphatidylinositol, mTOR, apoptosis, VEGF, focal adhesion, the regulation actin cytoskeleton, etc., and the complete details of the list of pathways for all these five genes are presented in **Table 2**. These genes presented in the networks (**Figure 3**) are known to play critical roles in a number of complex human diseases, including T2D and obesity, and the particulars have been presented in more detail in the *Discussion* section.

**Table 2:** Selected gene-specific pathways.

Gene	Pathways
SPNS1	T cell receptor signaling pathway Fc epsilon RI signaling pathwayNatural-killer-cell-mediated cytotoxicity
PTPN6	Adherens junction Jak-STAT signaling pathway Natural-killer-cell-mediated cytotoxicity T-cell receptor signaling pathwayB-cell receptor signaling pathway
CD247	Natural-killer-cell-mediated cytotoxicity T-cell receptor signaling pathway
FOS	MAPK signaling pathway Toll-like receptor signaling pathway T-cell receptor signaling pathway B-cell receptor signaling pathway TNF signaling pathway cAMP signaling pathway Osteoclast differentiation Circadian entrainment Estrogen signaling pathway Prolactin signaling pathway Oxytocin signaling pathway
PIK3R5	ErbB signaling pathway Chemokine signaling pathway Phosphatidylinositol signaling system mTOR signaling pathway Apoptosis VEGF signaling pathway Focal adhesion Toll-like receptor signaling pathway Jak-STAT signaling pathway Natural-killer-cell-mediated cytotoxicity T-cell receptor signaling pathway B-cell receptor signaling pathway Fc epsilon RI signaling pathway Fc gamma R-mediated phagocytosis Leukocyte transendothelial migration Neurotrophin signaling pathway Regulation of actin cytoskeleton Insulin signaling pathway Progesterone-mediated oocyte maturation Type II diabetes mellitus Aldosterone-regulated sodium reabsorption Bacterial invasion of epithelial cells

## Discussion

Diabetes mellitus (DM) and obesity are considered metabolic-disorder-related diseases, and DM is characterized by hyperglycemia resulting from defects in insulin secretion, insulin action, or both (1, 18, 32). Diabetic chronic hyperglycemia is associated with long-term damage, dysfunction, and failure of various organs such as the kidneys, eyes, nerves, blood vessels, and heart (1, 17, 19, 24, 32–34). T2D is most often found to be frequent in the case of obesity, and it is characterized by abnormal insulin secretion and/or a decreased sensitivity to insulin, which is also known as insulin resistance, and it results in increased blood glucose levels; T2D is known to be strongly associated with obesity (29). There are a number of biological processes and mechanisms that are considered to be the source of association between these conditions *via* mediating inflammation in adipose tissue and systemic insulin resistance, such as oxidative stress, endoplasmic reticulum stress, hypoxia, amyloid and lipid deposition, lipotoxicity, and glucotoxicity.

In this study, the obese samples totaled 36 samples where 18 were lean needle biopsy and 18 obese biopsy samples; these samples were age-matched to lean (n = 19) and obese (n = 18) female subjects, and the abdominal subcutaneous fat specimens have been collected. The average age (years) of the patients was 35.7 +/- 8.9 (obese-needle), 37.2 +/- 9.3 (obese-surgery), 37.2 +/- 9.9 (lean-needle), and 36.8 +/- 10.1 (lean-surgery), and the average body mass index (BMI) was 44.0 +/- 6.9 (obese-needle), 46.1 +/- 4.4 (obese-surgery), 21.9 +/- 1.9 (lean-needle), and 21.9 +/- 2.4 (lean-surgery) (35). While in the case of T2D, GSE121 (36) was used for gene expression profiling, which contains insulin resistance (IR) and insulin sensitivity (IS) for type II diabetes, while 11 were control samples, and the age of patients was not mentioned. The main goals of the authors (in the case of T2D) were to identify DEGs in skeletal muscle in insulin resistance, a major risk factor for T2D (non-insulin-dependent) diabetes, and to compare the gene expression patterns of skeletal muscle tissues from 18 insulin-sensitive versus 17 insulin-resistant, equally obese, and non-diabetic Pima Indians for which global transcript profiling has been performed (36). Different from the isolated study of obesity or T2D, our goal differed much and accomplished the following: presented a comparative study of both these two diseases; greatly explored the role of immune systems main TCR signaling pathway; and uniquely presented the network of immune signaling pathways and their components (**Figure 2B**).

With such levels of complexities, there exist a number of challenges for understanding the mechanism; we have therefore thoroughly analyzed the DEGs and have enriched pathways with the main focus on immune signaling pathways and the networks of DEGs belonging to the immune system. Among the top enriched pathways are the following: cell adhesion molecules, PI3K—AKT, MAPK, regulation of actin cytoskeleton, cAMP, phospholipase D, Focal adhesion, hematopoietic cell lineage, phagosome, Rap1, tight junction, leukocyte transendothelial migration, ECM-receptor interaction, and Ras signaling pathways.

For immune-system-related analysis, we observed that there are 10 pathways associated with the immune system in the case of T2D and 4 pathways in the case of obesity. Hematopoietic cell lineage and leukocyte transendothelial migration pathways are commonly altered in both the cases T2D and obesity and are known to play roles in blood cell development and immune surveillance and inflammation. Oxytocin and APP are obesity-specific immune signaling pathways and NK-cell-mediated cytotoxicity, insulin signaling, TCR signaling, ubiquitin-mediated proteolysis, platelet activation BCR signaling, neurotrophin, and oxytocin signaling pathways are T2D immune-system-specific pathways. Almost all these pathways (obese and T2D immune signaling pathways) are major parts of the immune system and are known to control major parts of the immune system. Furthermore, it is also known that altered TCR signaling is associated with T2D. Therefore, we have now mapped out the individual genes that are associated with the TCR signaling pathway and presented the networks for all the five genes (SPNS1, PTPN6, CD247, FOS, and PIK3R5), which are the components of the TCR signaling pathway, and their association with the other TCR genes (**Figure 3**). These five genes, SPNS1, PTPN6, CD247, FOS, and PIK3R5, are the potential components of TCR and also a number of additional immune-system-associated pathways. Among those pathways are NK-cell-mediated cytotoxicity, FC-epsilon RI signaling pathways, BCR, JAK-STAT, adherens junction, TLR, MAPK, TNF, cAMP, estrogen, oxytocin, prolactin, circadian entrainment, and osteoclast differentiation. PIK3R5 alone belongs to a large number of critical pathways such as TCR, BCR, NK cell-mediated cytotoxicity, JAK-STAT, ErbB, chemokine, phosphatidylinositol, mTOR, apoptosis, VEGF, focal adhesion, the regulation actin cytoskeleton, etc. (37). The SPNS1 gene is known to be associated with a number of human diseases, including T2D and obesity (38–42), which is mainly associated with lysosome storage disorder (LSD). PTPN6 is also known to be directly associated with controlling the inflammatory system, and altered PTPN6 (expression/mutation) may lead to potential human diseases (43–45). CD247 is known to regulate blood pressure, which is a critically affected factor in the case of T2D and obesity, by altering T-cell infiltration in the kidney; it also has an important impact on other human diseases (46–48). FOS is considered to play a role in inflammatory bone and skin disease (49, 50) and also in multiple other human diseases. PIK3R5 contributes to cell survival and is a critical pathway component to control many diseases (51–53).

As we can see in the results, PIK3R5 and FOS, which are the components of the TCR signaling pathway, control a large set of biological functions, most of which are a part of the immune system, and CD247, PTPN6, and SPNS1 comparatively control fewer pathways; in terms of connectivity, however, PTPN6 has the highest connectivity followed by SPNS1, CD247, FOS, and PIK3R5. It means that PIK3R5 simultaneously controls a large number of biological functions but does not impact more genes; in other words, the higher connectivity of the gene will increase the likelihood of affecting the expression patterns of the gene or vice versa. In terms of future perspectives, this study will be helpful for the development of therapeutic approaches and drug targeting.

## Materials and Methods

For obese people, we used the GSE12050 (35) dataset; 18 of 36 samples were lean needle biopsies, and these were compared with 18 obese biopsy samples. The samples were age-matched lean (n = 19) and obese (n = 18) female subjects. For sample details, it has been mentioned that abdominal subcutaneous fat specimens have been collected by using a needle biopsy approach from 9 obese patient samples and 10 lean samples. The average ages of the patients were 35.7 (obese-needle), 37.2 (obese-surgery), 37.2 (lean-needle), and 36.8 (lean-surgery). A total of nine obese patient samples were collected from abdominal subcutaneous fat biopsies and nine lean samples from abdominal fat biopsies (35). For T2D, GSE121 (GPL101 platform) (36) has been used for T2D gene expression profiling, which contains 5 IR and 5 IS samples with type II diabetes while 11 are control samples, and the age of patients have not been mentioned. The samples were non-diabetic Pima Indians or Tohono O’Odham Indians who were classified as IS or IR (36). For this dataset, the details and the goal submitted by the submitters were as follows: to identify DEGs in skeletal muscle in insulin resistance a major risk factor for T2D (non-insulin-dependent) diabetes and to compare gene the expression pattern of skeletal muscle tissues from 18 insulin-sensitive versus 17 insulin-resistant, equally obese, non-diabetic Pima Indians for which global transcript profiling has been performed (36).

These datasets were collected from GEO and have been processed for expression value calculation, fold change, and p-value calculation by the in-built code at GEO, i.e., GEO2R (54, 55). The list of DEGs has been processed for pathway enrichment analysis and understanding (56, 57). The KEGG (37) database has been used for calculating enriched pathways, and our own code has been used for pathway and network analysis (58–61). To retrieve the DEGs association as a network, the FunCoup2.0 network database (62) was utilized throughout the work, and the network was visualized with the help of cytoscapes (63). For most of our coding and calculations, MATLAB was used.

Now, we briefly summarize all the steps used are raw file processing, intensity calculation, and normalization. As the high-throughput data of the mainly microarray-based gene expression values are calculated as intensity ranging from the lowest to highest value, it is a necessity to have optimized or scaled-down values so we normally convert them to log2 values. For normalization (64–66), GCRMA (67–71), RMA, and EB are the most commonly used approaches. Here, we have used EB for raw intensity normalization. After normalization, we proceed towards our goal, which is to understand the gene expression patterns (56, 72) and their inferred functions (56, 57). For differential gene expression prediction and statistical analysis, MATLAB functions (e.g., mattest) have been used and volcano plot. From *mattest*, we get the p-values, and we then go for p-value corrections, assign the p-value threshold, and use the volcano plot for fold change calculation and the selection of genes with p-values less than 0.05 and fold changes within the range >= +2.0 and <= -2.0. This range of fold change along with this p-value cutoff are commonly used, while, in our study, we selected the top-ranked genes to have the strict condition of selection based on fold change so the fold changes are either much higher or lower than the cutoff fold change. For pathway analysis, we used the KEGG (37) database and have our own code designed for the pathway and network analysis (73–75). We have described the fold change, and, by considering genes as DEGs, we have applied thresholds for FC >= +2.0 (upregulated genes) and FC <= -2.0 (downregulated genes); these DEGs have been considered for pathway enrichment analysis. But for heatmap and network presentations, the top 100 DEGs (50 up and 50 down) genes have been presented (76, 77).

## Conclusions

Here, we observe that there are fewer genes that are commonly differentially expressed, while there are comparatively more pathways that are shared between them. There are only 4 pathways that are associated with the immune system in the case of obesity and 10 immune-associated pathways in the case of T2D, and, among them, only 2 pathways are commonly altered. Here, we have studied the differentially expressed genes (DEGs), enriched pathways, and the connection between immune signaling pathways *via* their components between obesity and T2D with a focus on the roles of the TCR signaling components. We conclude that in the case of T2D, there are more alterations in the gene expression pattern of those genes that are known to control the major parts of the immune system than in the case of obesity, where there is less of an effect than with T2D, but these few pathways are also known to be associated with controlling critical roles of the immune system.

## Data Availability Statement

The original contributions presented in the study are included in the article/**Supplementary Material**. Further inquiries can be directed to the corresponding authors.

## Author Contributions

Conceptualization, BE, HS, HC, and MM. Methodology, BE, HS, HC, and MM. Software, MM. Validation, BE, HS, HC, and ZZ. Formal analysis, BE, HS, HC, and MM. Investigation: BE and MM. Resources, BE and MM. Data curation, BE and MM. Writing—original draft preparation, BE, HS, HC, and MM. Writing—review and editing, BE, HS, HC, and MM. Visualization, BE, HS, HC, and MM. Supervision, HC and MM. Project administration, BE and HC. Funding acquisition, BE. All authors contributed to the article and approved the submitted version.

## Funding

This research was funded by the Deanship of Scientific Research (DSR) at King Abdulaziz University, Jeddah, grant number RG-5-140-39 and Swedish Cancer Society.

## Conflict of Interest

The authors declare that the research was conducted in the absence of anycommercial or financial relationships that could be construed as a potentialconflict of interest.

## Abbreviations

DEGs, Differentially expressed genes; TCR, T cell receptor; ISPs, Immune signaling pathways; T2D, Type-2 diabetes; APP, Antigen processing and presentation; APC, Antigen-presenting cell.
